# Methodological guidance on clinical prediction models in mental health research

**DOI:** 10.1017/S003329172610378X

**Published:** 2026-06-09

**Authors:** Raquel Costa, Bruno de Sousa, Thomas Kneib, Rui Martins, Andreas Mayr

**Affiliations:** 1 HEI-Lab: Digital Human-Environment Interaction Labs, Lusófona University, Campo Grande 376, 1749-024 Lisboa, Portugal; 2 Faculty of Psychology and Education Sciences, CINEICC, University of Coimbra, Coimbra, Portugal; 3 Chair of Statistics and Campus Institute Data Science, Faculty of Business and Economic Sciences, Georg-August-University of Göttingen, Göttingen, Germany; 4 Department of Mathematics, University of Coimbra, Coimbra, Portugal; 5 Institute for Medical Biometry and Statistics, Marburg University, Marburg, Germany

**Keywords:** depression, mental health, clinical prediction models, prognostic models, personalized carer, diagnosis, prognosis, external validation, data-driven approaches, predictive accuracy, machine learning, AI-driven decision

## Abstract

Clinical prediction models play a crucial role in advancing personalized care for mental health disorders, providing essential insights for diagnosis, prognosis and intervention planning. This work examines the current methodological approaches used to develop such models, emphasizing their application to mental health problems, including depression. To illustrate these concepts, we used data on prenatal depression from a multinational observational study of 5,372 pregnant women. The goal is to develop an individual prognostic model for depressive symptoms that can be used already at the beginning of pregnancy. Our analysis explores variable selection strategies, validation methodologies and the integration of clinical expertise with data-driven approaches. Particular attention is given to addressing challenges such as population heterogeneity, overfitting and the importance of external validation for generalizability across diverse settings. We distinguish between statistical regression models and machine learning techniques, discussing their respective strengths and limitations in terms of interpretability, predictive accuracy and clinical usability. This work offers practical guidance for researchers and clinicians, focusing on the critical steps for model development and implementation. We highlight best practices to avoid common pitfalls, advocate for interdisciplinary collaboration and address challenges of integrating advanced statistical and machine learning tools into clinical practice. By providing practical guidance and addressing these issues, our aim is to support the development of robust and clinically relevant prediction models.

## Introduction

With the rise of powerful statistical, machine learning (ML) and artificial intelligence (AI) techniques, as well as the availability of rich data sources in digital medicine, the use of prediction models for screening, diagnosing and planning personalized interventions in mental health appears to have great promise for both patients and healthcare providers. However, beneath this promise lies the current clinical reality: translating these innovative techniques into clinical practice in general, or even personalized mental healthcare in particular, remains a challenging endeavor (Markowetz, 2024). This work emphasizes the critical methodological aspects to consider during the planning or analysis of prediction models and offers recommendations for selecting the most appropriate techniques.

In general, one can distinguish between diagnostic and prognostic prediction purposes. Diagnostic models are based on the cross-sectional association between clinical data and an unknown outcome. Their aim is typically to evaluate the current state of the individual, either to determine whether symptoms or a disease are present or to quantify their severity to start or guide treatment. On the other hand, prognostic models aim to assess a longitudinal association, for example, to predict a future clinical outcome (Cook, 2008). Here, the aim could be to model the individual risk of developing a disease in the future, to predict a response to a treatment or also to predict the survival time of a patient.

**Figure 1.**
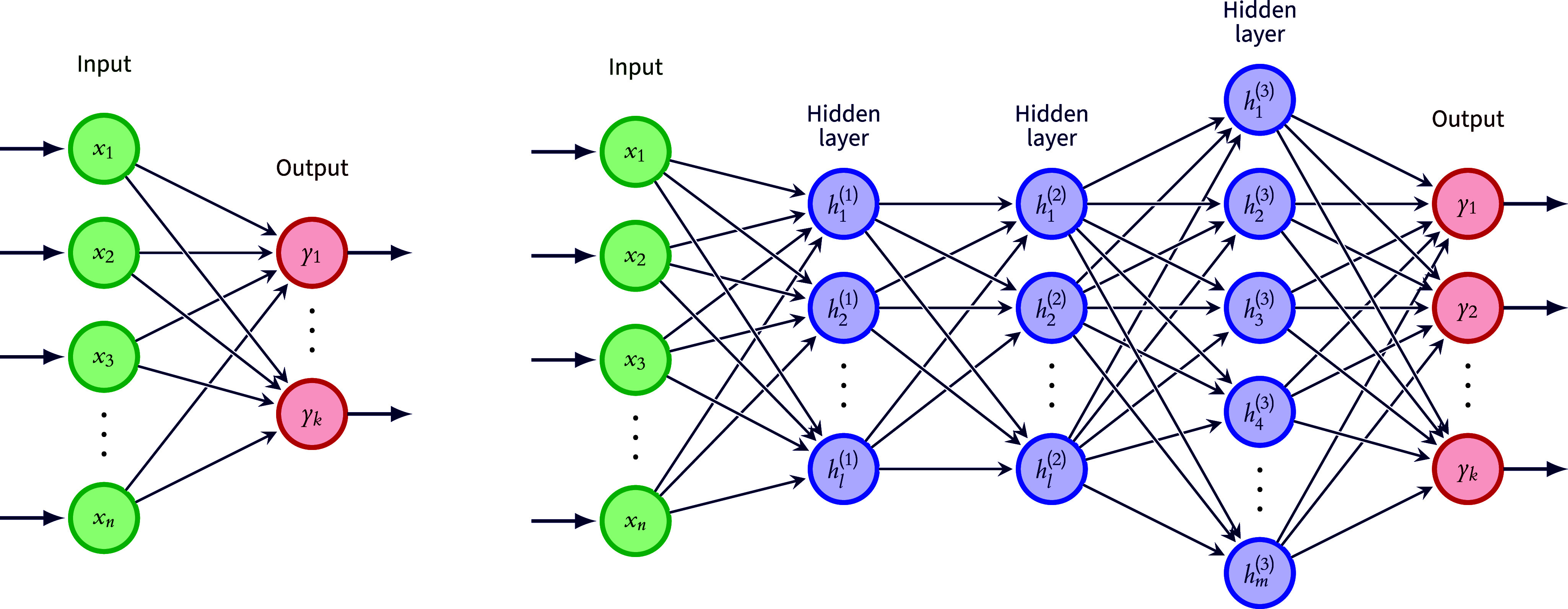
Scheme representing a regression (left) and a deep neural network with three hidden layers (right). Figure 1. long description.

To derive diagnostic and prognostic prediction models, one can use a variety of approaches ranging from classical statistical modelling to modern ML approaches and AI support systems. In this article, we distinguish between statistical techniques (e.g., penalized regression such as LASSO) that construct interpretable prediction models based on structured additive regression-type models and ML tools (e.g., tree-based approaches or deep neural networks) predominantly employed in computer science (Hastie, Tibshirani, & Friedman, 2009). Statistical techniques typically rely on structured models that allow for the interpretability of individual predictors or risk factors, while ML tools are less reliant on structural constraints and assumptions and may therefore produce improved predictions without providing readily interpretable effects of individual predictors or risk factors. For example, neural networks define hidden layers of nodes (also called neurons) connected to each other, which enables the model to learn complex patterns and interactions, but intertwining possible interpretations of risk factors. Statistical regression is a layer-free approach, producing predictions directly from inputs (see Fig. 1 for a contrast of these approaches). Of course, in practice, the separation between statistical approaches and ML is not always as clear-cut, and there are various approaches that are situated at the intersection of both fields or combining ideas from both worlds.

We will illustrate the methodological issues in the development of prediction models, considering multinational data on perinatal depression (Motrico et al., 2021). The aim of the illustrative example is to develop a prognostic model for depressive symptoms during pregnancy. The model should be able to be used by healthcare providers to screen the overall population of pregnant women at the beginning of pregnancy. We also emphasize that, in addition to methodological tools assisting the choice of a specific model, the selection of predictor variables, or the comparison of rival model specifications, it is important to involve clinical or practical expertise in the model-building process.

Although many of our points are relevant for all clinical prediction models, in this work, we will focus particularly on mental disorders. Mental disorders have been in recent years the target of various clinical prediction models (Lee et al., 2022; Ziobrowski et al., 2023). The Global Burden of Disease (GBD) Study 2019 reported that mental disorders are one of the top ten leading causes of disease burden worldwide, and among these, depressive disorders account for the largest proportion of mental disorder-related disability-adjusted life years (DALYs) in 2019 (37.3%, GBD 2019 Mental Disorders Collaborators, 2022). Depression is among the leading causes of years lived with disability (YLDs, James et al., 2018), and of health loss worldwide, responsible for 38.7 million DALYs, and there was a significant increase due to the COVID-19 pandemic (additional 10.7 million DALYs, reaching a total of 47.4 million DALYs), especially in women and young adults (Santomauro et al., 2021). Although screening for depression in primary healthcare settings is associated with a decrease in severity of symptoms and higher rates of remission of depression (O’Connor et al., 2023), underdiagnosis and undertreatment of mental health problems in general remain a global public health problem (Abuse and Administration, 2018; Ghafari, Nadi, Bahadivand-Chegini, & Doosti-Irani, 2022; Rens et al., 2022). About one in four (23%) of all adults and about half of young adults (18–29 years) report unmet needs for mental healthcare (European Union, 2022). In the perinatal period, the gaps are alarming, with only about one in ten women with clinically significant symptoms of depression receiving mental healthcare (Costa et al., 2024). In addition to the personal burden, untreated depression is associated with an important negative social impact in terms of productivity at work, regardless of the country’s economic characteristics (Evans-Lacko & Knapp, 2016). Therefore, the development and implementation of cost-effective tools that facilitate the identification of populations at risk are important for timely and adequate mental healthcare throughout the world.

The remainder of this article is structured as follows: In Section “A literature review of the current state,” we will present a short review of the literature on current practice in the development and validation of clinical prediction models for mental disorders. In Section “Illustrative example on prenatal depression,” we briefly introduce our illustration example, a prediction model for depression in the perinatal period. In Section “Methods to develop prediction models,” we then move toward statistical and ML tools that can be used to develop prediction models. In the following Section “General methodological issues and pitfalls,” we highlight general methodological issues and challenges that researchers face – no matter which techniques they use to estimate their model. In Section “From the model to the clinical decision,” we finally focus on aspects concerning the implementation of prediction models in clinical decision-making.

## A literature review of the current state

We have searched in PUBMED, during May 2023, for papers published in 2022 and 2023 using the search expression (“prediction” OR “supervised learning” OR “penalized regression” OR “calibration” OR “risk score”) AND (“mental health” OR “depression” OR “anxiety” OR “mental disorder”) and retrieved *n* = 118 papers. We excluded those not related to the topic of predicting symptoms of mental health problems (*n* = 96). The selection process followed the guidelines of the Preferred Reporting Items for Systematic Reviews (PRISMA), resulting in the retention of 22 articles. We analyzed the statistical methods used to predict mental health problems (Table 1), along with the population characteristics and study endpoints (Table 4). The included studies were conducted in the United States, Europe, China, Korea, United Kingdom, Brazil, Saudi Arabia, Denmark, Japan, Korea, China, Israel, Sweden and Canada. Sample sizes range from *n* = 87 to *n* = 39,439 participants. The region, sample size and age of the participants were not reported in some of the studies. Most use self-reported questionnaires to assess mental health outcomes and a binary (*n* = 15) or binary combined with continuous (*n* = 3) endpoint (Table 4).

**Figure 2.**
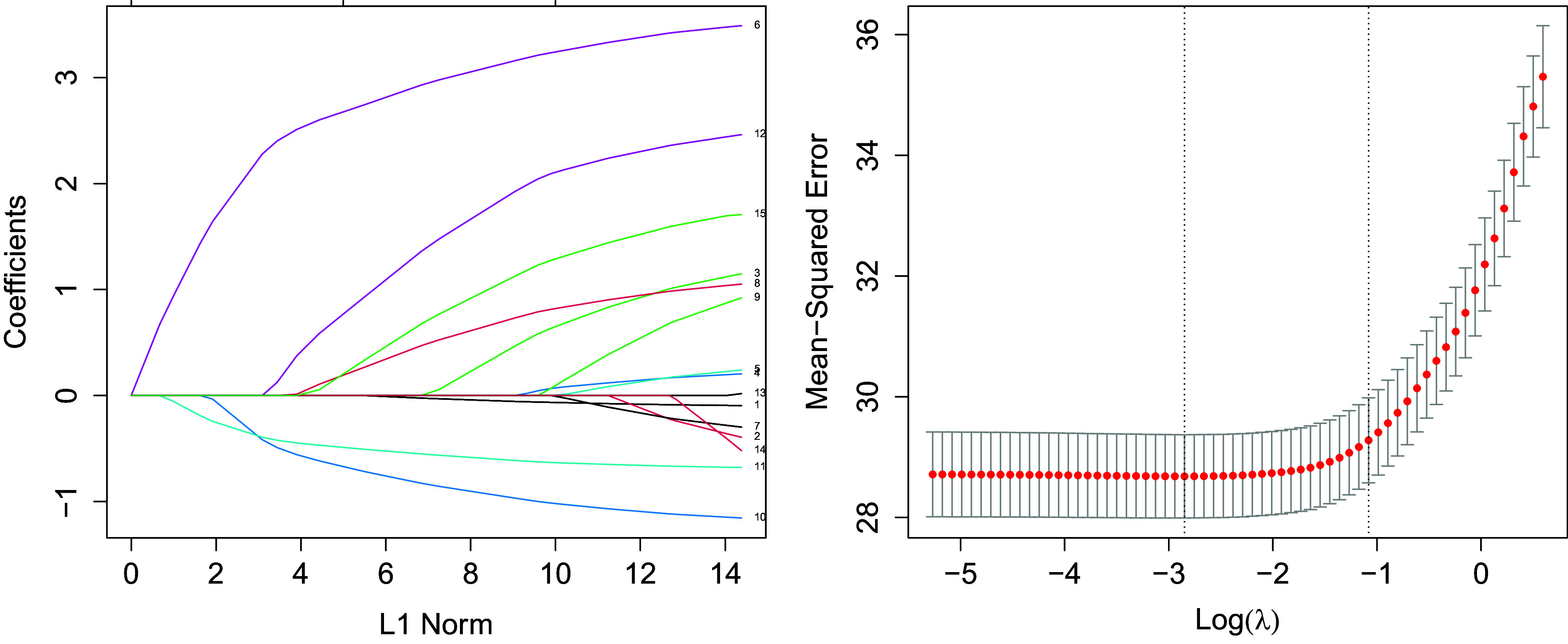
Coefficient path of a L1-penalized statistical model (LASSO, left) and the corresponding tuning of the shrinkage parameter *λ* (right) to avoid overfitting. Figure 2. long description.

**Figure 3.**
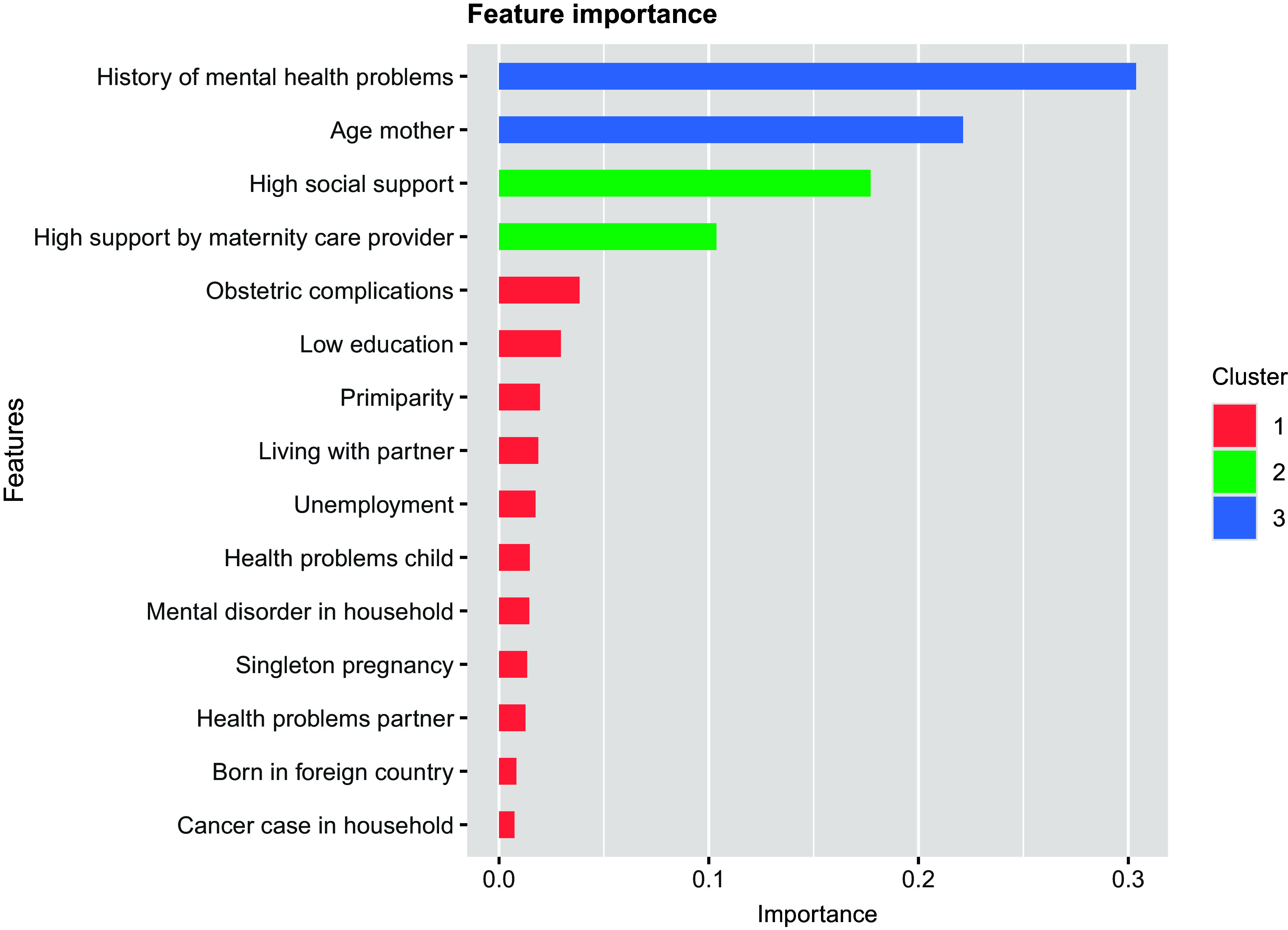
Variable importance plot to predict EPDS resulting from xgboost. The so-called *feature importance* is a relative measure, commonly used in ML, attributing the contribution of each variable to the performance of the prediction model. The colors refer to post-hoc cluster analysis on the importance values, in this case leading to three clusters of decreasing importance: blue represents the strongest predictors, the history of mental problems and the age of the mother. Figure 3. long description.

**Figure 4.**
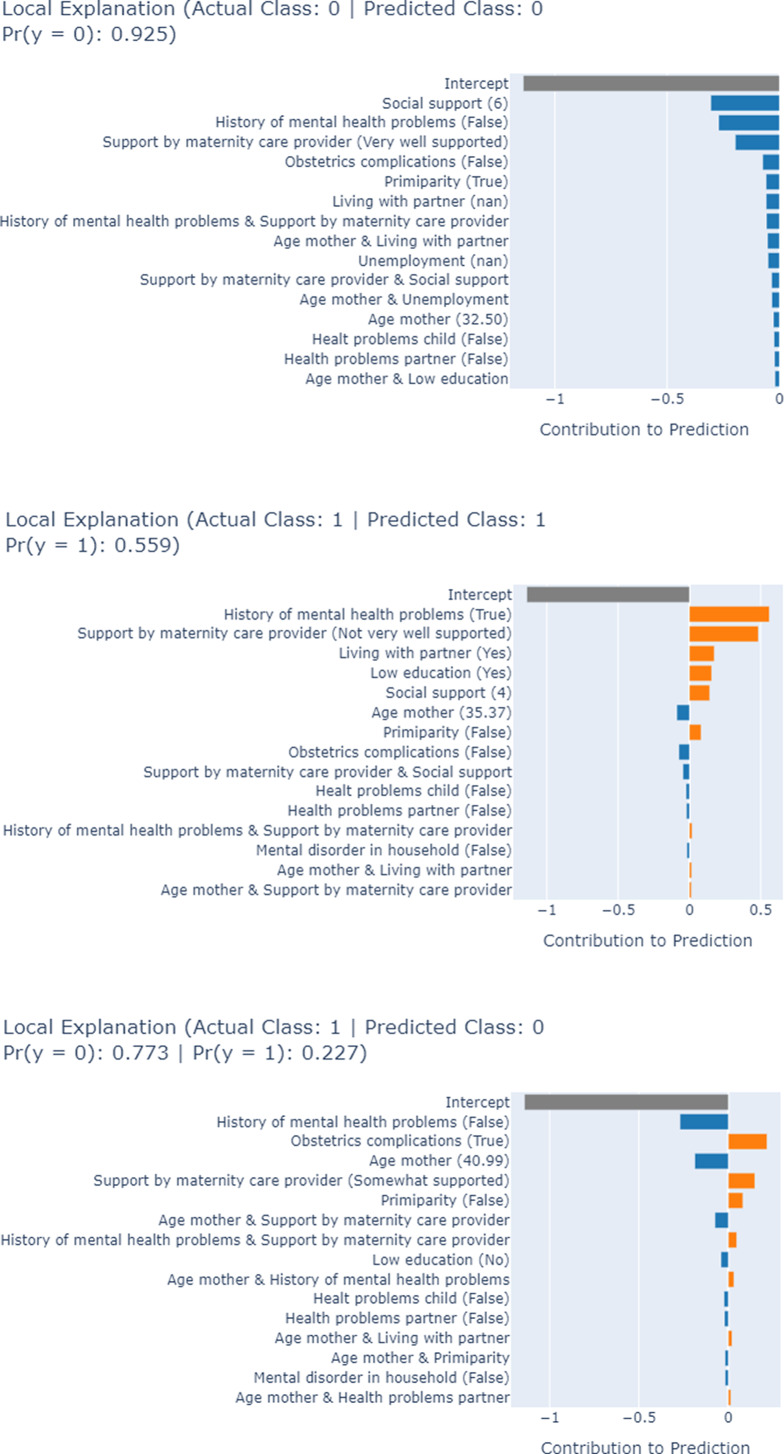
InterpretML: Three different prediction explanations of a woman’s classification: correctly predicting no depression (top), correctly predicting depression (middle) and incorrectly predicting no depression (bottom). Note that “nan” means information not available. Figure 4. long description.

**Table 1.** Literature search results: overview on methods and approaches for fitting, selecting and validating a prediction model for mental illnesses Table 1. long description.

	Articles (*n* = 22)	%
**Methods for prediction**		
Regression	17	77
Tree-based	10	45
Neural-network	5	23
Ensemble of above	3	14
**Variable selection**		
Data-driven	13	59
Prior knowledge	11	50
Not described	2	9
**Validation**		
Single-split (internal)	7	32
Cross-validation (internal)	9	41
External	4	18
Not described	5	23

Note: Many articles use multiple methods, therefore the percentages do not sum up to 100%.

For building prediction models, most studies (77%) employed some form of regression analysis, while others used decision tree-based methods or artificial neural networks. The methods and approaches for fitting, selecting and validating a prediction model for mental disorders used in these studies are shown in Table 1. To select the predictor variables, around half of the studies applied some kind of data-driven variable selection approach while the remaining focused on variables that – according to previous publications – are assumed to be informative for the outcome (e.g., prior knowledge). To validate the prediction performance, only 4 of the 22 studies relied on external data. Most studies performed internal validation (*n* = 9 cross-validation, *n* = 7 single-split). Particularly worrying is the observation that a notable proportion of publications on prediction models (*n* = 5 studies, 23%) do not report validation on separate observations, which is alarming from a methodological perspective since predictive ability will then likely be overestimated (see Section “Development and validation cohort”).

## Illustrative example on prenatal depression

### Data

Data were collected within the scope of the RISEUP-PPD-COVID-19 cross-sectional study (ClinicalTrials.gov registration: NCT04595123) conducted in 13 countries (Albania, Argentina, Brazil, Bulgaria, Chile, Cyprus, Greece, Israel, Malta, Portugal, Spain, Turkey, United Kingdom) with the aim of assessing and determining the risk and protective factors for perinatal mental health problems during the COVID-19 pandemic. A detailed description of the methods is available in Motrico et al. (2021). The sample consists of adult pregnant and postpartum women (with biological children up to 6 months of age). From these, participants who were pregnant and completed the Edinburgh Postnatal Depression Scale (EPDS, Cox, Holden, & Sagovsky, 1987), as well as data on the predictors of interest described later in the article, were included for the illustrative example (*n* = 5,372).

### Variables

Symptoms of depression were assessed using the EPDS, a 10-item self-report scale. The total scores is calculated through the sum of the scores obtained in each item, with total scores ranging from 0 to 30. Higher scores indicate a higher severity of symptoms. Potential predictors of depression symptoms were chosen based on a literature review, and the probability that the data can be available in some healthcare facilities or easily obtained already during pregnancy (see Section “Variable selection”). Some of the variables in the database were not included in the model due to chronological constraints (see Section “Prognostic vs. diagnostic models”), since they were exclusive for women in the postpartum period (*n* = 16; e.g., low birth weight at birth) or because, according to the available literature, they were not considered relevant for the prediction of depression (*n* = 52; e.g., owning or renting the residence). The variables included sociodemographic information, including age, country of residence, country of birth (country born vs. foreign born), educational level (secondary education or lower vs. higher educational level), unemployment (yes vs. no); obstetric-related data, including gravidity (primipara vs. multipara), type of pregnancy (single vs. multiple), obstetric complications (yes vs. no); mental health related data, including history of mental health problems (yes vs. no), current treatment for mental health problems (yes vs. no); medical conditions of close relatives, including history of medical conditions of partner or child (no vs. yes, if any of the following – respiratory disease, diabetes, heart disease, lung disease, liver disease, cancer, disease compromising the immune system, mood and/or anxiety disorder), history of cancer of close relatives (i.e., partner, child, other relative) living in the same house hold (no vs. yes), history of mental disorders of close relatives (i.e., partner, child, other relative) living in the same household (no vs. yes); and healthcare and social support information, including perception of the quality of support by the primary maternity care provider (scale from 1 – not very well supported – to 3 – very well supported), and the perception of support by the social network (scale from 1 – not supported – to 7 – highly supported).

### Aim of the prognostic model

The underlying objective of the prognostic model is to assess the individual liability of a woman at the beginning of pregnancy to develop symptoms of prenatal depression (PND). The outcome will be the EPDS score. The model should be constructed in a way that healthcare providers can use it to screen the general population of pregnant women, relying ideally on information that could be available in clinical records (e.g., without the need for additional contacts to the individual) or easily obtained already during pregnancy. The focus on accessible clinical data sources and valid measures at the individual level is recommended for improving prediction models (Kraus, Sampathgiri, & Mittal, 2024).

### Software and Data

All data cleaning, transformations and statistical modeling for this study were performed using the statistical computing environment R (R Core Team, 2024) and Python (Python Software Foundation, 2025). For modeling approaches, different add-on packages like glmnet, mboost, xgboost and InterpretML were used. The code to reproduce our analyses is available at PrognosticModel.git. Due to considerations of data privacy and confidentiality, the original data cannot be made publicly available. However, we provide an artificial dataset with similar characteristics, which can be used for reproducibility.

## Methods to develop prediction models

### Statistical prediction models

When developing a clinical prediction model, the decision for a specific model type depends on, inter alia, (i) the type of the outcome variable that shall be predicted (e.g., continuous vs. binary), (ii) the available explanatory information (e.g., number of covariates; types of covariates), (iii) specifics of the data collection process (grouping arising, e.g., from multicenter studies; designed experiment vs. observational data). In any case, regression models form the most prominent class of statistical models usually employed to develop prediction models due to their interpretability and the broad software availability to estimate them. In the following, we discuss some important decisions for specific types of regression models.

Concerning the type of outcome variable, linear models for continuous, approximately Gaussian responses, logistic regression for binary outcomes and Poisson regression for count responses are more often used despite a plethora of other types of models that have been developed in statistics. Especially for continuous responses, it is quite common to apply transformations to the responses to take restrictions on the domain of the response into account (e.g., a logarithmic transformation for non-negative responses) or to make the distribution of the responses more symmetric (Fahrmeir, Kneib, Lang, & Marx, 2022, Ch. 3). Although an exact match with the normal distribution is not as central to a prediction model as it is to a model from which statistical conclusions on model parameters should be performed (confirmatory analyses), transformations that turn the distribution of the response into something close to a normal are often also beneficial for prediction quality. In practice, the normal distribution often serves as a useful starting point for analyzing quasi-continuous scores. In our analysis, we hence consider the EPDS score as the primary response variable and treat it as a quasi-continuous outcome.

For covariates, the classical challenge is to determine/select which of them are relevant for the prediction problem at hand (see Section “Variable selection”). Expert knowledge, for example, derived from a literature review or clinical experience, can often guide the decision of a suitable set of potential covariates, but data-driven variable selection is usually applied in an additional step. The most typical cases are stepwise selection approaches based on some model performance criterion such as Akaike’s information criterion (AIC) (Cavanaugh & Neath, 2019), but alternatives such as LASSO regression (Zhang, Wei, Lu, & Pan, 2020) or boosting methods (Mayr, Binder, Gefeller, & Schmid, 2014) have attracted increasing interest. In addition to the ideal subset of covariates, transformations of covariates may be considered in the variable selection step. The general aim is to determine a model that achieves good predictive abilities (i.e., it should include all relevant covariates for the prediction task) but is also easily applied in practice (i.e., it should contain a small set of variables that are directly available).

In our illustrative analysis, we rely on 15 candidate variables that could be available from clinical records or easily obtained already during pregnancy, mostly reflecting socioeconomic and clinical characteristics. Afterward, we will then perform data-driven variable selection via penalized regression with the LASSO. Table 2 reports the resulting coefficients for our example on PND, while Fig. 2 displays the corresponding coefficient paths and the tuning of *λ.* The LASSO method was able to perform an automated variable selection. The effect estimates of variables with minor importance are shrunken toward 0, which means that they are effectively excluded from the model. The tuning parameter *λ* controls the selection of variables and the shrinkage of effect estimates; larger values of *λ* lead to sparse models (strong regularization), and small values of *λ* lead to larger models close to a classical regression model without regularization. In our case, *λ* was optimized via a 10-fold cross-validation (re-fitting the model and checking for prediction performance). Of the 15 candidate variables, 9 are selected to be part of the prediction model. The direction of the effects is as expected, for example, a *history of mental problems* has a strong positive effect on the predicted EPDS.

**Table 2.** Resulting coefficients of a penalized regression model for EPDS, fitted via the LASSO Table 2. long description.

	coefficient
(Intercept)	13.53
Age mother	−0.03
Born in foreign country	
Living with partner	
Low education	0.31
Unemployment	
History of mental health problems	2.89
Primiparity	−0.09
Obstetric complications	0.70
Singleton pregnancy	
High support by maternity care provider	−1.01
High social support	−0.57
Health problems child	0.84
Health problems partner	
Cancer case in household	
Mental disorder in household	0.34

*Note:* Variables without entry have been shrunk toward 0, and are effectively excluded from the model (see also Fig. 2 for the corresponding coefficient paths). For a comparison to the variable importance from a ML approach, see Fig. 3 Note that country was not considered as a variable here, in order to ensure also predictions to regions not included in our data are possible.

Especially when combining data from multiple trials or multiple centers that collected the data, it can be important to control for unobserved heterogeneity in the trials/centers. As a consequence, random effects models for the different centers are typically employed in such contexts where the random effects are representatives for the unobserved heterogeneity (Tutz, 2026). In transnational studies, random effects can also be used to represent country-specific heterogeneity. Similarly to the distributional assumption for the response discussed earlier, the inclusion of random effects is less essential for a prediction model than for a confirmatory analysis of a clinical trial, where they are often considered a means of adequately reflecting uncertainty. However, considering unobserved heterogeneity and confounding effects can be relevant to ensure that the prediction model can be transferred across different contexts. For a more detailed look at the challenge of generalizability of prediction models, see Section “Generalizability.”

In any kind of regression model, the regression effects provide interpretable information on the contribution of the different covariates toward the prediction, which is useful both for clinicians themselves and for the communication with patients (for a recent discussion of different model types, see Heller, 2024). In our EPDS example, one can observe in Table 2 for example a small negative effect of the *age* of the mother, meaning that we predict a lower EPDS score (less depressive symptoms) for older women. On the other hand, a *history of mental problems* is estimated to have a strong positive effect: considering everything else equal, a woman who had suffered from mental problems in the past is predicted to experience a 2.89 points higher EPDS score. For comparison, 10 years of age only reduce the predicted EPDS by 0.3. Note that the LASSO estimated model incorporates variable selection (some coefficients are set to 0) and does not directly provide confidence intervals for coefficients. Nevertheless, prediction models are usually built for prediction purposes such that quantification of uncertainty for the estimated coefficients is of lesser relevance than in other contexts.

### Machine learning and algorithmic predictions

ML has emerged as a modern way of constructing prediction models that often achieve better predictive performance compared to classical statistical regression models (as discussed in the previous section) at the price of lacking interpretability (Lucasius et al., 2025). More precisely, many ML approaches do not make strong structural assumptions about the impact of covariates on the outcome of interest, but rather “let the data speak” to determine the most suitable model specification. This includes the automatic consideration of interactions between covariates that are usually restricted in statistical models (or at least have to be included manually). Despite these different goals of statistical models and ML, there is also considerable overlap in terms of approaches that are – depending on the community – considered to belong to the one or the other field. For example, LASSO regularization is sometimes claimed to be part of ML but originated in the field of statistics (Caner, 2021).

One particularly prominent example of ML prediction models is random forests relying on an ensemble of trees (classification or regression trees, depending on the type of the response variable, Breiman, 2001; Hastie, Tibshirani, & Friedman, 2009). Trees are based on iteratively splitting the data set based on the covariates available such that the resulting partitioning makes partitions homogeneous with respect to the response of interest while maximizing between-partition heterogeneity. Each partition is then assigned a predicted outcome. Due to the iterative splitting approach, trees are very flexible and also include interactions between covariates, but are typically quite unstable, that is, the tree might change considerably in response to even quite small changes in the data. Random forests compensate for this by considering an ensemble of trees built on random subsets of the data, which increases the stability of the approach. The individual covariate effects are then of complex form and only hardly accessible, but covariates can still be assigned a variable importance to facilitate a certain level of interpretability.

Another typically tree-based approach is gradient boosting. In contrast to random forest that grow trees independently on bootstrap samples, gradient boosting grows them sequentially on re-weighted observations. Observations that have been misclassified before receive higher weights (Freund & Schapire, 1996) A very powerful and fast implementation is XGBoost. Note that there are also statistical boosting algorithms, which are based on a similar principles but yield statistical models (Bühlmann & Hothorn, 2007; Friedman, Hastie, & Tibshirani, 2000).

In many applications, ML approaches achieve very good prediction accuracy and can outperform classical statistical models in this respect. However, in addition to the more challenging interpretation, they are also more difficult to turn into clinical practice since they cannot be easily represented in a formula/equation that can be evaluated in day-to-day practice. In addition to random forests, other forms of ML could also be used to construct prediction models. This includes approaches such as support vector machines (Song and Diederich 2013) but also neural network approaches, establishing a connection to the area of AI (see Section “Challenges and opportunities in AI-driven decision support systems”).

In Fig. 3, we display the variable importance estimates from a tree-based approach – extreme gradient boosting (XGBoost – (Chen & Guestrin, 2016) to estimate an algorithmic solution. In contrast to the LASSO solution in Table 2, we cannot directly interpret the model itself, in the sense of interpreting the direction and size of effects via coefficients, but we need to assess the relative importance of the variables for the prediction performance to gain some insights (*feature importance*). The ML model was run on the same set of candidate variables. Also here we can see a strong importance of the *history of mental problems* variable, followed by the *age* of the mother. Most of the variables that had not been selected by the LASSO are also showing a very low importance.

### Challenges and opportunities in AI-driven decision support systems

AI has revolutionized various sectors, and mental healthcare is no exception. Decision Support Systems (DSS) powered by AI could, in the future, provide better prognostic and diagnostic accuracy, personalized treatment plans and improved patient outcomes. These systems are designed to help clinicians make informed decisions, thereby optimizing mental healthcare delivery.

Traditional diagnostic methods are partially based on subjective assessments, which can lead to misdiagnosis or overlooked conditions. Recent work of Bradford et al. (2024) clearly calls for more research to identify missed opportunities in the diagnosis of mental health problems. AI-driven DSS analyzes large amounts of data from various sources, including clinical records, patient interviews and even social media activity, to identify indicative patterns of specific mental health disorders. As mentioned in Section “Machine learning and algorithmic predictions,” ML algorithms as well as big data analytics can be used to process this volume of data and detect subtle signs and symptoms that might be missed by human clinicians. For example, Natural Language Processing (NLP) algorithms can use patient speech or writing to analyze signs of depression, anxiety or other mental health conditions. Although these data-driven approaches tend to produce more accurate and timely diagnoses, ultimately leading to better patient outcomes, they lack interpretability, transparency and robustness. These are some of the challenges identified in recent studies by Higgins, Short, Chalup, and Wilson (2023) and Andrew, Rudra, Eunice, and Belfin (2023) that can justify the small number of studies considered in the integrated review by Higgins, Short, Chalup, and Wilson (2023).

To address these challenges, Nori, Jenkins, Koch, and Caruana (2019) proposed InterpretML, an open source Python package designed as a unified framework for ML interpretability. It offers two approaches to interpretability: Glassbox and Blackbox forms. The Blackbox model provides explanations for any ML model, regardless of its complexity, using interpretation techniques such as LIME, SHAP and Partial Dependence Plots. The Glassbox models are ML algorithms designed to give clear interpretations of the decisions provided for models such as linear models, decision trees and generalized additive models (GAM). It uses an Explainable Boosting Machine (EBM) algorithm, an extension of the GAM that allows the automatically inclusion of pairwise interactions, providing two levels of explanations: global and local effects. Thus, InterpretML extends linear models such as LASSO regression approaches beyond linearity, opening the black-box models such as ML approaches to inspection and interpretability, where visual plots are provided to show how the different covariates affect predictions, allowing intuitive interpretation to non-technical decision-makers.

Consider our EPDS data set with the 15 variables previously presented as predictors and a dependent variable defined as 1 (depression) if a woman’s EPDS score was 13 or higher, and zero (no depression) otherwise.

Local explanations are instance-specific and provide insight into why a particular prediction was made for a specific input. In Fig. 4, we clearly see that the main factors that contribute to a no-depression classification (top) are high social support, no history of mental problems, strong support from the maternity health provider and no obstetrics complications. For a diagnosis of depression (EPDS ≥13), it is worth noting the inclusion of living with a partner and low education at the top of the list (middle). Finally, for a misdiagnosis of no depression (bottom), the most contributing factor is the lack of a history of mental problems and being older (41 years). Note also that the estimated probability of depression in this case is 0.227, high enough for the healthcare professional to consider a potential case of developing depression.

Although the potential promise of AI-driven DSS in reducing the burden and burnout of medical professionals is immense, giving time back to health professionals, Bossewitch et al., 2022 alert to the possible harm of the use of algorithmic and data-driven technologies in the mental health context, which could lead to further stigmatization of patients, overdiagnosis and unnecessary medication (Thakkar, Gupta, & De Sousa, 2024).

## General methodological issues and pitfalls

### Preprocessing data

A well-known saying in the community with respect to data is: “garbage in, garbage out…”. If the dataset is of low scientific quality, that is, non-representative, ambiguous, noisy, imbalanced or very sparse, this might likely translate into bad or inaccurate findings, no matter the quality or the sophistication of the model. Therefore, effective data preprocessing is important for any data analysis. The goals and characteristics of the research question and the requirements of the models will drive the preprocessing procedures. These procedures are also sometimes summarized under the term *initial data analysis* (Baillie, Le Cessie, Schmidt, & Lusa, 2022; Heinze et al., 2024). This might include verifying the distribution of the variables, checking for inconsistencies or suspicious patterns in responses to identify or filter out unreliable data (e.g., checking for outliers or missing responses). In addition, one of the most common preprocessing techniques involves the transformation of variables. Transformations are used for various reasons, primarily to correct skewness or to standardize the data by centering and scaling. Many predictive models perform better when the predictors are standardized on a common scale. However, a significant drawback is that the interpretability of the estimated effects can be lost because the data are no longer in their original units. For instance, predictive models based on decision trees (Section “Statistical prediction models”) are less sensitive to the characteristics of predictors. In contrast, classical linear regression (Section “Machine learning and algorithmic predictions”) heavily depends on these characteristics.

Some of the data collected in mental health surveys are on an ordinal scale, for example, perception of the quality of support by the primary maternity care provider (1 – not very well supported; 2 – well supported; 3 – very well supported), which means that the response points are ordered, but the distance between the values is without meaning. Unlike nominal data, these variables/features have a distinct rank or order, adding complexity to the preprocessing steps. Often, they need to be encoded to facilitate their inclusion in predictive models by assigning numerical values to categories based on their order. It is crucial to ensure that the assigned numerical values respect the inherent order of the categories, which means that higher values must correspond to higher ranks or levels on the ordinal scale. For example, perception of the quality of support by the primary maternity care provider might be coded as 0 – not very well supported; 1 – well supported; 2 – very well supported. Alternatively, an ordinal (monotonic) encoding can be applied, where the ordered categories are transformed into integers that reflect their ranking without assuming equal distances between them; this approach allows models that explicitly handle ordinal predictors (e.g., ordinal logistic regression or tree-based methods with monotonic constraints) to incorporate the ordering information directly. A dummy variable encoding can also be used when order is not relevant, but each category needs to be represented distinctly, by assigning a binary indicator (1 or 0) to each category.

Data eligibility is another challenge. In our PND example, data were collected online (for more information on methods, see Motrico et al., 2021); therefore, special attention was paid to making sure that all participants were, in fact, complying with the eligibility criteria and that there were no duplicates in the database. Since in online platforms there is no guidance from a researcher, it is quite often that participants misunderstand the information on eligibility criteria or answer more than once, especially when the dissemination of the study is ongoing for several months. To manage these challenges, several approaches can be implemented. For instance, in our illustrative example, we checked the questions regarding the eligibility criteria and excluded participants who did not meet the criteria. The questionnaire was anonymous, so to check for potential duplicates, we looked at the information on the date of birth of the participants and the child. In case there was a match of dates, we used other sociodemographic variables to ascertain the likelihood of being the same person. In cases of high matches, we disregarded the second entry in the questionnaire and considered only the first (Mesquita et al., 2023).

### Missing data

Among all the obstacles to start the construction of a predictive model, missing data is likely one of the most challenging. It is of utmost importance to understand why the values are missing, because this will drive the researcher’s decision about what to do with the predictors or with the respective participants for whom some values are missing. After flagging the missing values, one is typically faced with two options. Simply remove individuals or variables with missing values or try to fill in the gaps in the data set (imputation). If the pattern/characteristics of the missing values somehow allow us to anticipate the outcome of the response, then we will be in the presence of what is commonly called an informative omission (Little & Rubin, 2019, Ch. 13). A typical example of this situation is when, during a follow-up, some patients stop attending depression therapy sessions, thus stop reporting their depression levels. These dropouts may be due to significant improvement of symptoms, making them feel that reporting is unnecessary. This is noted in many research studies on attrition in mental health (Cuijpers et al., 2014; Warden et al., 2007). Statistically, this situation is especially challenging because researchers cannot simply exclude individuals with missing data; the absence of a measurement at a specific time point can, in itself, reveal insights about the response. To address this, researchers could either develop an imputation model to predict the missing values or use the missingness as a separate category (see, e.g., Figure 4). We refrain from going further into this subject, as this is beyond the scope of our work. For a comprehensive review and an evaluation of the methods dealing with missing data can be found elsewhere (Carpenter et al., 2023; Graham, 2009; Van Buuren, 2018; Wang & Ware, 2013).

### Prognostic vs. diagnostic models

In both statistical and ML literature, a model that aims to predict an unknown or unobserved outcome *y* based on variables or features *x* is typically called predictive. This prediction of the outcome does not necessarily relate to a future observation, but can also be just an unknown characteristic. However, in the medical literature, the process of assessing a current unknown status of a patient is typically called *diagnostic* (Van Smeden et al., 2021). Many current applications of deep learning or AI techniques in medical imaging, such as radiology, are based on cross-sectional data on the association between predictor variables and outcome (Hosny et al., 2018; Secinaro et al., 2021). These are, therefore, by their nature, diagnostic, as the disease is already present (and can be detected in the corresponding images). In the case of prognostic models for future outcomes, the association between predictor variables and the outcome is longitudinal, and the objective is to assess the individual risk of a patient for a future occurrence of a disease (Collins et al., 2015).

In the case of our example on PND, this distinction can be easily illustrated by the underlying aim of the prediction model. If the goal is prognostic, the prediction model should identify women at high risk of developing depression in the future, allowing preventive measures to be initiated. From a preventive point of view, the crucial time point for applying the prognostic model could be the beginning of pregnancy, to allow early identification and intervention (Curry et al., 2019; RISEUP-PPD Guidelines Development Group, 2022). If the aim was diagnostic, the model should identify pregnant women who are currently suffering from depression, allowing timely clinical diagnosis and treatment.

This distinction significantly influences the selection of potential predictor variables. In cases involving longitudinal associations, such as in prognostic modeling, it is essential to respect the chronological order of events. For example, in our model of PND, only variables available in clinical records or easily obtained at the beginning of pregnancy should be considered, as that is the intended time for prognosis. In our example, derived from a large international observational study with extensive data collected throughout pregnancy, adhering to this chronological constraint drastically reduces the pool of potential predictor variables for a prognostic model (see Section “Variables”).

In contrast, a diagnostic model for depression can incorporate any available variable to determine whether a woman is currently experiencing depression. For instance, in our dataset, the most informative covariate for EPDS is Generalized Anxiety Disorder-7 (GAD-7), a widely used scale to assess anxiety symptoms that is strongly correlated with EPDS. However, since GAD-7 is measured at the same time as EPDS, it is suitable for a diagnostic model but not for a prognostic model.

It is evident that prognostic modeling presents a more challenging task in practice, as the longitudinal nature of associations may be subject to change over time (Cook, 2008).

### Variable selection

One of the most critical decisions in the development of a clinical prediction model is the selection of the predictor variables that will form the model (Collins et al., 2015). This decision has an important impact on the accuracy achievable by the final model, as only a combination of highly informative variables can build a valid prediction model. However, the choice of the variable set also has very relevant consequences for the applicability of the model and its translation into clinical practice.

Clinical prediction models will only be implemented in real-world settings if the predictor variables underlying them are readily accessible to clinical decision-makers or can be easily and quickly assessed. To develop a prediction model for eventual use in clinical practice, after external validation, it is therefore essential not only to focus on selecting the set of predictors that yield the highest possible performance but also to bear in mind that healthcare professionals who will later employ this model will need access to all these data.

In our example on PND, this means that if the objective is to build a model for internal screening by healthcare providers to identify women at risk, the subset of predictor variables that could form the model should consist of information easily available to healthcare providers. For example, variables in questionnaires such as anxiety symptoms, while highly informative about depression, should not be included in the final model. By considering the chronology of events (as discussed in Section “Prognostic vs. diagnostic models”) and selecting only variables that could be available or easily obtained at the beginning of pregnancy, the number of potential predictors is reduced to only 15 of the more than 83 variables available in the data set.

In practice, it is sensible to pre-filter existing variables regarding their availability. This can be viewed as a subject-matter-driven variable selection (Heinze, Wallisch, & Dunkler, 2018; Staerk, Byrd, & Mayr, 2024). Such filtering or also the complete variable selection may also rely on prior subject matter knowledge, such as known risk factors or findings from the literature (see Table 1). In contrast, many methods to estimate prediction models (see Section “Methods to develop prediction models”), incorporate some kind of data-driven variable selection. Optimizing the corresponding tuning parameter with respect to prediction accuracy directly affects the model complexity (either via a shrinkage parameter, the number of iterations of an algorithm, or an information criteria).

It is important to note that the selection of the most suitable set of variables is not automatically the same as identifying the most important risk factors for the event or outcome of interest (Shmueli, 2010). The aim of a prediction model is to find the combination of factors leading to the highest prediction accuracy, taking into account their interdependence. In our example on PND, we therefore used a ML or statistical approach to select and estimate the best combination of the 15 potential predictor variables (see also “Section “Variables””).

### Metrics and measures to evaluate prediction models

An essential step after developing a prediction model is to assess its accuracy (Steyerberg, 2019). This assessment should be performed using a separate validation cohort that was not used during model development or parameter estimation (see Section “Development and validation cohort”). But how can one determine whether a model is accurate? The choice of evaluation metric depends on the specific prediction target and the clinical question being addressed. For example, in the case of continuous outcomes, such as the EPDS in our illustrative example, the focus might be on point predictions (e.g., the severity of a disease as measured by the EPDS or the value of a specific medical marker) or on predicting reference ranges for specific populations (e.g., typical biomarker values for a given age group).

For binary outcomes or classification problems (e.g., EPDS ≥13 vs EPDS <13), the primary interest is often in predicting individual probabilities (e.g., the risk of developing clinically significant depressive symptoms). These probabilities can subsequently be categorized into discrete labels, such as high-risk or low-risk individuals, for practical application.

For both types of responses – binary and continuous – different evaluation metrics are commonly used. These metrics can be broadly categorized into those assessing a model’s calibration and those evaluating its discrimination (Cook, 2008). Calibration refers to how well the predicted values align with the observed outcomes (Van Calster et al., 2019). This is often assessed graphically using calibration plots, which compare observed values with predicted values or probabilities (e.g., observed vs. predicted probabilities, as described in Collins et al., 2015). In contrast, discrimination reflects a model’s ability to distinguish between individuals with different outcomes, such as correctly identifying those at higher versus lower risk (Pencina, D’Agostino, & Vasan, 2008).

For binary outcomes, the most commonly used discriminatory measures are the AUC, as previously mentioned, and the more general C-statistic, both of which range from 0.5 (no discrimination) to 1 (perfect discrimination). For continuous outcomes, a commonly used discriminatory measure is *R*^2^ for test data, which represents the proportion of variance in the outcome explained by the model (Staerk, Klinkhammer et al. 2024). Some metrics evaluate both calibration and discrimination (Steyerberg, 2019). For example, the Brier score for binary outcome measures the mean squared difference between observed outcomes (0 or 1) and predicted probabilities (Brier, 1950). For continuous outcomes, the analogous measures are the Mean Squared Error of Prediction (MSEP) and its square root, the Root Mean Squared Error of Prediction (RMSEP).

### Development and validation cohort

To assess the accuracy of prediction models, irrespective of whether they are prognostic or diagnostic, it is extremely important to validate the performance on unseen observations that have not been included in the model development, e.g., by utilizing an external validation cohort (Collins et al., 2024). Evaluating the model on already seen observations would most likely lead to drastically overoptimistic results and heavily biased conclusions about the abilities of the model.

Unfortunately, in some of the prediction models we found in our literature review, it cannot be ruled out that this issue is present (see Table 1), as no separate validation set was described. In this context, one could assume that models are trained toward overfitting, a problem often encountered in prediction models (Hastie, Tibshirani, & Friedman, 2009; James, Witten, Hastie, & Tibshirani, 2013). In order to minimize the discrepancy between model and observations, classical fitting approaches (e.g., maximum likelihood procedures) for statistical models will estimate the best model for the development cohort. As a result, the model focuses too much on peculiarities in this underlying sample and performs poorly on observations outside this sample. Ignoring the distinction between in-sample and out-of-sample performance by validating a model on already seen observations from the development cohort makes the identification of overfitting behavior impossible.

To overcome potential biases and ensure a fair assessment of a prediction model, external validation is considered the gold standard. Ideally, the model should be tested on entirely separate data. This requires validation carried out by different researchers, in different regions or hospitals, to ensure robustness in various settings.

A second-best option is so-called temporal validation, where patients recruited until a specific time point constitute the development cohort, while those recruited afterward form the validation cohort. This procedure attempts to mimic external validation and typically leads to more realistic results than just randomly splitting the data (Collins et al., 2015).

Randomly splitting the data once or multiple times, through techniques such as bootstrapping, cross-validation or subsampling, constitutes internal validation. These methods are widely used in practice, as confirmed by our literature search. Internal validation (e.g., via bootstrapping) is designed to correct for optimism in apparent model performance and can provide approximately unbiased estimates of predictive performance for future individuals drawn from the same underlying population as the development cohort. However, when model performance is intended to generalize to different settings (e.g., routine clinical practice and not a clinical trial), populations (other regions, countries) or time periods (prognostic models), performance estimates obtained through internal validation may still be optimistic. This reflects the fact that internal validation does not account for differences in case mix, clinical practice or other forms of unmeasured heterogeneity between development cohort and target populations.

In clinical practice, dependencies and associations may often differ slightly from those observed in the trial used to develop the model. In order to report performance measures of the new model that are closer to clinical reality, it may be favorable to try to obtain truly external observations (or at least temporally splitted data) even if this leads to poorer estimated performances.

In our example on PND this can be easily illustrated due to multinational structure of the survey. We fitted linear prediction models for the EPDS via different estimation approaches (Table 3), including a step-wise variable selection via the AIC (Heinze, Wallisch, & Dunkler, 2018), a component-wise gradient boosting approach with linear base-learners (Bühlmann & Hothorn, 2007) and also a LASSO *L*_1_-regularized model (Tibshirani, 1996). We performed different splits of the data into development and validation cohorts. First, we simply randomly split the data (70/30 random split) before we performed a 10-fold cross-validation. Additionally, we performed a temporal split by including all cases before November 2020 in the development cohort and all after this time point in the validation cohort. Afterward, we also used a regional split by including all observations from Western + Central European countries in the development cohort and all others (South America, Israel, Turkey) in the validation cohort.

**Table 3.** Root mean squared error of prediction (RMSEP) evaluated on different test cohorts performing random splitting, 10-fold cross-validation, temporal split (before and after November 2020), and regional split: Western + Central European countries form the development cohort, all others (South America, Israel, Turkey) form the validation cohort Table 3. long description.

	Step-wise	Boosting	LASSO
Random split	5.31	5.31	5.40
10-fold CV	5.37	5.37	5.42
Temporal Split	5.44	5.42	5.45
Regional split	5.49	5.49	5.60

**Table 4.** Population characteristics and endpoints of the studies Table 4. long description.

Study	Population-sample size	Population-age	Population-region	Endpoints
Adler, Wang, Mohr, & Choudhury (2022)	*n* = 61 patients in treatment; *n* = 48 university students	Mean age of patients 37 years, not reported for students	USA	EMA, continuous
Albagmi et al. (2022)	*n* = 3,017	More than half aged between 20 and 39 years	Saudi Arabia	GAD–7, binary and categorical
Bjertrup, Væver, and Miskowiak (2023)	*n* = 87 pregnant women	Mean age 33 years	Denmark	MINI, binary; EPDS, continuous
Caye et al. (2022)	*n* = 1,072	19 years*	USA	YAPA, binary
Chavanne et al. (2023)	*n* = 156 participants with anxiety, *n* = 424 control group	18–23 and 14 (controls) years	Not reported	GAD–7, binary
Chung & Teo (2023)	*n* = 955	Not reported	Not reported	Mental health problems, binary
Cunha et al. (2023)	*n* = 1,442	Mean age 18 years*	Brazil	DAWBA, binary
Geffre, Carr, Meynadasy, & Sachs-Ericsson (2023)	*n* = 571	Over 60 years	USA	COVID–19-related distress, continuous
Kim, Park, & Kang (2022)	*n* = 116 potential depression participants, *n* = 80 control group	Mean age 59 and 49 (controls) years	Korea	BDI, binary
Liu et al. (2022)	*n* = 16,384	Over 18 years	UK	PHQ–9, continuous
Matsuo et al. (2022)	*n* = 10,013	Mean age 31 years	Japan	EPDS, Binary
Murri et al. (2022)	*n* = 39,439	Over 55 years	Europe	EURO-D, Binary
Noroña-Zhou et al. (2023)	*n* = 1,389 mother–child dyads	Mean child age 9 years	USA	SCARED, continuous and binary; CDI–2, continuous and binary
Östberg & Nordin (2022)	Population-based data	18–79 years	Sweden	HADS, continuous and binary
Qasrawi et al. (2022)	*n* = 3,984	10–15 years	Israel	GAD–7, binary; DSRS, binary
Roseberry et al. (2023)	*n* = 721	Not reported	Not reported	SAS–4, binary; STAI, binary
Sajjadian et al. (2023)	*n* = 192	Mean age 35.4 years	Canada	MADRS, continuous and binary
Wang et al. (2023)	*n* = 13,293	Mean age 54 years	USA	PHQ–9: binary
Wy et al. (2022)	*n* = 129	Not reported	Korea	PHQ–9, binary; GAD–7, binary
Yang et al. (2022)	*n* = 981	Mean age 31 years	China	EPDS, binary
Zhou & Reiter (2010)	*n* = 2,574	Over 18 years	China	PHQ–9, binary
Zhu (2022)	College students	Not reported	Not reported	Depression, binary

*Note*: USA, United States of America; EMA, Ecological Momentary Assessments; GAD-7, General Anxiety Disorders; MINI, Mini-International Neuropsychiatric Interview; EPDS, Edinburgh Postnatal Depression Scale; YAPA, Young Adult Psychiatric Assessment; DAWBA, Development and Well-Being Behavior Assessment; COVID-19, coronavirus disease 2019; PHQ-9, Patient Health Questionnaire-9; EURO-D, European Union initiative; SCARED, Screen for Child Anxiety Related Emotional Disorders; CDI-2, Children’s Depression Inventory, Second Edition; DSRS, Depression Self-Reported Scale; SAS-4, Simplified Anxiety Scale; MADRS, Montgomery and Åsberg Depression Rating Scale; *age at endpoint assessment.

It is notable that the difference in performance between the modelling methods is smaller than the different prediction errors achieved from different validation techniques: The root RMSEP is ranging from 5.31 to 5.60 for the different validations, see Table 3. Regarding the modelling methods, for example, for the temporal split, the difference is comparably marginal (5.42 to 5.45). This might be also due to the limited number of variables that we have to predict depression (Buckman et al., 2023), which limits the abilities of the more advanced methods compared to the step-wise variable selection for logistic regression. The regional split, which is closest to a real external validation, leads to the poorest results (5.49–5.60). However, these results might be the most realistic ones. The single random split of the data, representing internal validation, leads to the best (5.31–5.40) but perhaps overoptimistic results, which might be heavily influenced by high variability.

### Generalizability

While Section “Development and validation cohort” focused on validation strategies to assess predictive performance, an equally important challenge is the ability of prediction models to generalize beyond the specific conditions under which they were developed. Generalizability refers to the extent to which a model maintains acceptable performance across different populations, settings and time periods. This section therefore focuses on structural and design-related factors that influence generalizability and cannot be fully addressed through validation alone.

The most fundamental issue is that the development cohort covers a broad range of different settings that are diverse and representative of various clinical or population realities to which the model will later be applied. In our example on PND, where our objective is to develop a screening tool for pregnant women, the development cohort should cover the true heterogeneity of the general population. However, many cohorts from research studies tend to be biased toward participants with higher education and lack representation from individuals with low socioeconomic backgrounds. This also holds for our development sample (Motrico et al., 2021).

Additionally, to avoid overfitting in the first place, many statistical or machine-learning techniques are designed not to achieve the best fit on the development cohort but to provide good predictions on external observations and robustness to shifts in association structures. This is achieved by using penalization techniques, early stopping or careful tuning of hyperparameters based on prediction performance (Friedrich et al., 2023).

Prominent examples of the failure of prediction models to generalize well beyond the initial populations, particularly with respect to mental disorders, are genetic prediction models. These models are usually developed based on large cohort data or genome-wide association studies and aim to report the genetic predisposition of an individual toward a phenotype or trait. While these types of prediction models have made significant progress in recent years, their generalizability remains a substantial challenge. Typically, the underlying cohorts consist mostly of individuals with European ancestry, due to the dominance of genotyping performed in North American and European countries. Current research suggests that the performance of these models on individuals from non-European ancestries, which are underrepresented in the development cohorts, is very limited. One provocative study (Curtis, 2018) even concluded that a polygenic prediction model for schizophrenia was more strongly associated with ancestry than with schizophrenia. The reasons for this include different linkage (associations) and the prevalence of risk alleles in ancestry-based populations. Very current research tries to overcome these issues by incorporating methods from causal inference (Rothenhäusler & Bühlmann, 2023; Rothenhäusler, Meinshausen, Bühlmann, & Peters, 2021).

In the context of our illustrative example on PND, we benefit from a highly diverse dataset that includes observations from different countries, populations and sociodemographic backgrounds. However, there is a bias toward higher educated women with a better sociodemographic profile. This is most likely due to the self-selection process of the participants. In addition, there are significant differences between countries and regions. The EPDS threshold defining clinically significant symptoms varies across countries and languages. However, a threshold of 13 or more is often used to indicate clinically significant symptoms of depression, as recommended for a cut-off point with higher specificity in the perinatal period based on data from several countries, including seven of the countries in our analysis (Brazil, Chile, Greece, Malta, Portugal, Spain, Turkey and the UK; Levis et al., 2020). In the remaining countries included in our analysis, no cutoff point is mentioned in Argentina, Cyprus and Israel (Alvarado, Jadresic, Guajardo, & Rojas, 2015; Kandel-Katznelson, Maisel, Zilber, & Lerner, 2000; Lopez-Janer, Quiros, & Cabestrero, 2021). Furthermore, our analysis of different validation approaches (Table 3) highlights the limited generalizability of models developed in one set of countries when applied to others, underscoring the role of population-specific factors. A solution to this might be to develop different regional or population-specific prediction models.

## From the model to the clinical decision

After a prediction model was developed, the next challenge is to derive a clinical decision from the output.

In many situations, clinicians will expect to use thresholds to identify patients that need intervention. In this context, especially when using a prediction model for diagnostics or screening, one typically faces a trade-off between sensitivity and specificity of the model when choosing such a threshold. Making a model more sensitive, so that it detects many individuals with a certain disease, usually also increases the risk of false positives such that the specificity goes down and vice versa. Unfortunately, there is no easy way out of this dilemma, and the ideal compromise has to take into account the specific application problem (Hajian-Tilaki, 2018; López-Ratón, Rodríguez-Álvarez, Cadarso-Suárez, & Gude-Sampedro, 2014). Theoretically, optimal solutions can be derived by assigning costs to false positives and false negatives, but to estimate these costs is highly challenging in a medical context.

But also after a threshold was derived, or when the output of the model is directly used as a severity score, deriving clinical decisions and implementing them in practice is still a challenge. Regarding our EPDS example, a screening model that can be also applied without contacting women might be especially relevant in countries or facilities in which EPDS or similar tools are not available for systematic screening. The use of validated and standardized screening tools such as EPDS, one of the most frequently validated tools with good predictive validity for depression (Park & Kim, 2023; Rondung et al., 2024), at least once during the perinatal period, is recommended by the American College of Obstetricians and Gynecologists to provide important information on severity of symptoms and guide referral to perinatal mental healthcare (American College of Obstetricians and Gynecologists, 2018; El-Den et al., 2022). However, recommendations on timing, frequency or dedicated healthcare provider differ, and evidence on its effectiveness for systematic screening purposes in healthcare facilities is lacking, even in high-income countries (El-Den et al., 2022). Furthermore, although widely used internationally, EPDS is in many languages not available nor validated, and clinical cut-offs vary from culture to culture (Kozinszky & Dudas, 2015) or are directly not available. In this case or when there are no resources to conduct systematic perinatal mental health screening programs, clinical prediction models can be applied using patient clinical records to assess the level of risk and help healthcare providers make informed personalized decisions regarding perinatal mental healthcare needs. Thresholds could specified for symptom severity scores based on existing local data, literature and healthcare facility policies. If only higher EPDS scores are considered for referral (for example, cut-off value of 13 determined by maximizing specificity, Levis et al., 2020), only the most severe cases are addressed, leaving many women in need of mental healthcare untreated (false negatives). On the other hand, considering scores with the best combined sensitivity and specificity, that is, implementing a cut-off value of 10 or 11 (Levis et al., 2020; Rondung et al., 2024) for referral, will result in many unnecessary cases referred to mental healthcare, which would have disposable financial costs. The approach of a healthcare facility to screening and referral is of the highest importance but depends on a balance of available resources and expected benefits, with major implications for the short and long-term well-being of women, children and families.

With the rise of modern technologies and digital medicine, there is also hope for further improvement in clinical decision-making: Automated decision-making tools can have an important role in assisting clinicians by enhancing the efficiency of assessments and providing transparent data/model-driven insights toward more personalized and accurate medical care (Kelly et al., 2019). As mentioned in Section “Methods to develop prediction models,” this is still challenging because many data-driven options lack interpretability and transparency. ML approaches can yield accurate prediction but are difficult to translate into clinical decision making due to a lack of interpretability. To make resulting models explainable, they often provide global feature importance to compare variables among each other, or also (as in Fig. 4) they can display for each individual which factors lead to a particular prediction or decision.

Integration of AI in mental healthcare should complement, not replace, human clinicians, guaranteeing a transparent and free of bias process. This can only be achieved with the implementation of collaborative teams throughout the process, including technology developers to mental health professionals or, as suggested by Higgins, Short, Chalup, and Wilson (2023), with glass-box designs such as InterpretML (Nori, Jenkins, Koch, & Caruana, 2019), which give clinicians control and understanding of different decision-makings in mental healthcare. Furthermore, human oversight by healthcare professionals is warranted to ensure reliable decisions and patients safety (Haselager et al., 2024; Langer, Baum, and Schlicker 2024).

Reporting and critical evaluation of AI-based predictive model studies are also of the utmost importance, and extensions of existing guidelines such as TRIPOD (Transparent Reporting of a multivariable prediction model of Individual Prognosis Or Diagnosis) and PROBAST (Prediction model Risk Of Bias Assessment Tool) by Collins et al., 2021 are needed to adequately address the specific challenges posed by AI methods. These are the possible key features to developing and implementing patient-centered approaches in mental healthcare (Thakkar, Gupta, & De Sousa, 2024), allowing health professionals to make informed decisions in the diagnosis and intervention for their patients.

## Conclusion

This article provided information on the current methodological approaches used to develop prediction models in mental health and practical guidance on common pitfalls and points to consider using an illustrative example of PND. Unlike other health complications that can arise during the perinatal period (e.g., diabetes, preeclampsia), mental health problems are highly associated with the sociodemographic characteristics and clinical background of the individual. Therefore, prediction models based on this information, which may already be available in clinical records or can be easily obtained during pregnancy, can be extremely useful for screening women in contexts where reliable dedicated tools are not available or when there are no resources to conduct systematic perinatal mental health screening programs.

From a brief review, we have shown that most studies developing prediction models on perinatal mental health problems reported the use of classical statistic regression analysis without relying on external data to validate the model. We have illustrated how this can be problematic from a methodological point of view, using multicountry data from pregnant women to develop a prognostic model for depressive symptoms during pregnancy. Comparing the use of internal versus external validation, we have shown that the use of internal validation data (e.g., bootstrapping, cross-validation) may lead to overoptimistic results whereas an external validation (e.g., temporal or regional splitting) cohort resulted in a more realistic estimation of the prediction accuracy.

We have alerted to important methodological decisions and challenges to consider while developing models (type of models, variable selection, validation, generalizability, clinical utility) with a focus of mental health research. For more general recent guidelines for the development of clinical prediction models and step-by-step tutorials, we refer to Collins et al. (2024), Collins et al. (2024) and Efthimiou et al. (2024).

The literature shows that many prediction models developed in research settings fail to transition into clinical practice (Kelly et al., 2019; Moons et al., 2012; Reilly & Evans, 2006). Authors have frequently highlighted that many of the models published each year are rarely implemented in real-world healthcare settings (Wyatt & Altman, 1995). The reasons are varied, including methodological limitations such as insufficient external validation, poor accuracy or inadequate calibration. Additionally, some models are designed using sophisticated methods but fail to address clinical priorities, the specific decision points where they are needed or the characteristics of the available data at those moments (Markowetz, 2024).

To overcome these challenges, a paradigm shift is needed. Strong, multidisciplinary collaboration between clinicians, statisticians, data scientists and other experts is essential to bridge the gap between technical rigor in model development and clinical usability. Beyond technical considerations, prediction models must be developed with a deep understanding of the healthcare environment, including the resources, workflows and constraints of clinical practice.

Finally, incorporating transparent, interpretable methods and fostering trust among healthcare professionals are key to adoption. The integration of patient input can also ensure that models align with patient-centered care principles. Future efforts should focus not only on model performance but also on implementation strategies, usability, and the evaluation of their impact on health outcomes in diverse settings. By addressing these aspects, we can ensure that prediction models evolve from theoretical constructs to valuable tools that improve patient care and inform clinical decision-making.

## Data Availability

Due to considerations of data privacy and confidentiality, the original data cannot be made publicly available. However, we have generated an artificial dataset with similar characteristics, which can be used for reproducibility: https://github.com/RUIMgithub/PrognosticModelMentalHealthPregnantWomen.git. The code used for this analysis is also available from the same repository.

## Figure 1. Long description

On the left, the regression diagram shows green input nodes labeled x sub 1 to x sub n, each with arrows pointing directly to red output nodes labeled y sub 1 to y sub k. On the right, the deep neural network diagram starts with green input nodes x sub 1 to x sub n, each connecting to the first hidden layer of blue nodes labeled h sub 1 super 1 to h sub m sub 1 super 1. Arrows connect these to the second hidden layer of blue nodes h sub 1 super 2 to h sub m sub 2 super 2, then to the third hidden layer h sub 1 super 3 to h sub m sub 3 super 3. All hidden layers are fully connected. The final layer consists of red output nodes y sub 1 to y sub k, each receiving arrows from all nodes in the last hidden layer. Each panel is labeled with ‘Input’, ‘Hidden layer’, and ‘Output’ as appropriate.

Navigate back to Figure 1..

## Figure 2. Long description

The left panel is a line graph with x-axis labeled L1 Norm from 0 to 14 and y-axis labeled Coefficients from negative 1 to 3.5. Multiple colored lines represent individual model coefficients, each starting near zero and diverging as L1 norm increases. Some coefficients increase, others decrease, and several remain near zero, illustrating variable selection and shrinkage in LASSO regression. Each line is labeled with a number at the right end. The right panel is a line graph with x-axis labeled Log open parenthesis lambda close parenthesis from negative 5 to 0 and y-axis labeled Mean-Squared Error from 28 to 36. Red dots represent mean-squared error values for different lambda values, with vertical gray error bars. The curve is flat at lower lambda values, then rises sharply after log lambda equals negative 2, indicating increased error and overfitting as regularization decreases. Two vertical dashed lines mark the optimal lambda range for model selection.

Navigate back to Figure 2..

## Figure 3. Long description

The chart displays feature importance for predicting E P D S, with the x-axis labeled Importance ranging from 0.0 to 0.3 and the y-axis labeled Features. From top to bottom, the features are: History of mental health problems, Age mother, High social support, High support by maternity care provider, Obstetric complications, Low education, Primiparity, Living with partner, Unemployment, Health problems child, Mental disorder in household, Singleton pregnancy, Health problems partner, Born in foreign country, and Cancer case in household. Bars are colored by cluster: blue for Cluster 3 (History of mental health problems, Age mother), green for Cluster 2 (High social support, High support by maternity care provider), and red for Cluster 1 (all remaining features). The two longest bars are History of mental health problems and Age mother, both in blue, indicating the highest importance. The next two, in green, are High social support and High support by maternity care provider. The remaining features have shorter red bars, indicating lower importance. The legend at the right matches colors to clusters 1, 2, and 3.

Navigate back to Figure 3..

## Figure 4. Long description

From top to bottom, each panel is a horizontal bar chart labeled ‘Local Explanation’ with actual and predicted class, and probability values. The x-axis is ‘Contribution to Prediction’ ranging from negative one to positive one. The y-axis lists features such as ‘Intercept’, ‘Social support’, ‘History of mental health problems’, and combinations like ‘Age mother and Living with partner’. In the top panel, actual and predicted class are both zero, with most bars extending left, indicating negative contributions, especially ‘Social support (6)' and ‘Support by maternity care provider (Very well supported)'. In the middle panel, both actual and predicted class are one, with most bars extending right, indicating positive contributions, notably ‘History of mental health problems (True)' and ‘Support by maternity care provider (Not very well supported)'. In the bottom panel, actual class is one but predicted class is zero, with mixed bar directions; ‘History of mental health problems (False)' and ‘Obstetrics complications (True)' contribute positively, while ‘Support by maternity care provider (Somewhat supported)' and ‘Low education (No)' contribute negatively. Some features are marked as ‘nan’ for missing data. Each panel visualizes how individual features influenced the model's prediction for that case.

Navigate back to Figure 4..

## Table 1. Long description

The table has three columns: method or approach, number of articles, and percentage. The first section, methods for prediction, lists regression with 17 articles (77 percent), tree-based with 10 (45 percent), neural-network with 5 (23 percent), and ensemble of above with 3 (14 percent). The second section, variable selection, lists data-driven with 13 articles (59 percent), prior knowledge with 11 (50 percent), and not described with 2 (9 percent). The third section, validation, lists single-split internal with 7 articles (32 percent), cross-validation internal with 9 (41 percent), external with 4 (18 percent), and not described with 5 (23 percent). Note states that many articles use multiple methods, so percentages do not sum to 100.

Navigate back to Table 1..

## Table 2. Long description

Starting from the top row, the left column lists variables and the right column shows their coefficients. The intercept is 13.53. Age mother has minus 0.03. Born in foreign country and Living with partner are excluded, with no coefficient shown. Low education is 0.31. Unemployment is excluded. History of mental health problems is 2.89. Primiparity is minus 0.09. Obstetric complications is 0.70. Singleton pregnancy is excluded. High support by maternity care provider is minus 1.01. High social support is minus 0.57. Health problems child is 0.84. Health problems partner and Cancer case in household are excluded. Mental disorder in household is 0.34. Variables without coefficients are shrunk toward zero and excluded from the model.

Navigate back to Table 2..

## Table 3. Long description

From the top row downward, the table lists test cohorts: Random split, 10-fold C V, Temporal Split, and Regional split. For each cohort, root mean squared error of prediction values are given for Step-wise, Boosting, and LASSO methods. Random split: Step-wise 5.31, Boosting 5.31, LASSO 5.40. 10-fold C V: Step-wise 5.37, Boosting 5.37, LASSO 5.42. Temporal Split: Step-wise 5.44, Boosting 5.42, LASSO 5.45. Regional split: Step-wise 5.49, Boosting 5.49, LASSO 5.60. Regional split defines Western plus Central European countries as the development cohort, and South America, Israel, Turkey as the validation cohort.

Navigate back to Table 3..

## Table 4. Long description

Beginning at the top row, the table presents studies in the leftmost column: Adler, Wang, Mohr, and Choudhury (2022) with 61 patients in treatment and 48 university students, mean patient age 37 years, region USA, endpoint EMA continuous. Next, Albagmi et al. (2022) with n equals 3,017, over half aged 20 to 39 years, region Saudi Arabia, endpoint G A D dash 7 binary and categorical. Bjertrup, Vae ver, and Miskowiak (2023) with n equals 87 pregnant women, mean age 33 years, region Denmark, endpoints MINI binary and E P D S continuous. Caye et al. (2022) with n equals 1,072, age 19 years, region USA, endpoint Y A P A binary. Chavanne et al. (2023) with n equals 156 participants with anxiety and 424 controls, ages 18 to 23 and 14 years, region not reported, endpoint G A D dash 7 binary. Chung and Teo (2023) with n equals 955, age and region not reported, endpoint mental health problems binary. Cunha et al. (2023) with n equals 1,442, mean age 18 years, region Brazil, endpoint D A W B A binary. Geffre, Carr, Meynadasy, and Sachs Ericsson (2023) with n equals 571, age over 60 years, region USA, endpoint COVID dash 19 related distress continuous. Kim, Park, and Kang (2022) with n equals 116 potential depression participants and 80 controls, mean ages 59 and 49 years, region Korea, endpoint B D I binary. Liu et al. (2022) with n equals 16,384, age over 18 years, region UK, endpoint P H Q dash 9 continuous. Matsuo et al. (2022) with n equals 10,013, mean age 31 years, region Japan, endpoint E P D S binary. Murri et al. (2022) with n equals 39,439, age over 55 years, region Europe, endpoint E U R O dash D binary. Noro n a dash Zhou et al. (2023) with n equals 1,389 mother child dyads, mean child age 9 years, region USA, endpoints S C A R E D continuous and binary, C D I dash 2 continuous and binary. Ostberg and Nordin (2022) with population based data, ages 18 to 79 years, region Sweden, endpoints H A D S continuous and binary. Qasrawi et al. (2022) with n equals 3,984, ages 10 to 15 years, region Israel, endpoints G A D dash 7 binary, D S R S binary. Roseberry et al. (2023) with n equals 721, age and region not reported, endpoints S A S dash 4 binary, S T A I binary. Sajjadian et al. (2023) with n equals 192, mean age 35.4 years, region Canada, endpoint M A D R S continuous and binary. Wang et al. (2023) with n equals 13,293, mean age 54 years, region USA, endpoint P H Q dash 9 binary. Wy et al. (2022) with n equals 129, age not reported, region Korea, endpoints P H Q dash 9 binary, G A D dash 7 binary. Yang et al. (2022) with n equals 981, mean age 31 years, region China, endpoint E P D S binary. Zhou and Reiter (2010) with n equals 2,574, age over 18 years, region China, endpoint P H Q dash 9 binary. Zhu (2022) with college students, age and region not reported, endpoint depression binary. Abbreviations in the table footnote include U S A for United States of America, E M A for Ecological Momentary Assessments, G A D dash 7 for General Anxiety Disorders, MINI for Mini International Neuropsychiatric Interview, E P D S for Edinburgh Postnatal Depression Scale, Y A P A for Young Adult Psychiatric Assessment, D A W B A for Development and Well Being Behavior Assessment, COVID dash 19 for coronavirus disease 2019, P H Q dash 9 for Patient Health Questionnaire dash 9, E U R O dash D for European Union initiative, S C A R E D for Screen for Child Anxiety Related Emotional Disorders, C D I dash 2 for Children’s Depression Inventory Second Edition, D S R S for Depression Self Reported Scale, S A S dash 4 for Simplified Anxiety Scale, M A D R S for Montgomery and Asberg Depression Rating Scale. Age at endpoint assessment is marked with an asterisk.

Navigate back to Table 4..
